# The effect of drying temperature of milk thistle seeds on quality and bioactive compounds in the lipid fraction

**DOI:** 10.1007/s13197-020-04431-4

**Published:** 2020-04-15

**Authors:** Sylwia Marszałkiewicz, Aleksander Siger, Marzena Gawrysiak-Witulska, Dominik Kmiecik, Magdalena Rudzińska

**Affiliations:** Faculty of Food Science and Nutrition, Poznań University of Life Sciences, Wojska Polskiego 28, 60-637 Poznan, Poland

**Keywords:** Drying, Phytosterols, Fatty acids, Tocopherols, Milk thistle

## Abstract

Milk thistle oils are available on the market and appeal to consumers because of their healthy properties as cold-pressed oils. The raw material for producing such oils is purchased from a range of domestic and foreign sources. The aim of this work was to determine the effect of drying temperature on the peroxide value, acid value, fatty acid composition, tocopherol and phytosterol contents in the lipid fraction extracted from milk thistle seeds. The seeds were purchased in three different farms and were dried in a thin layer at 40 °C, 60 °C, 80 °C, 100 °C, 120 °C, and 140 °C. The level of phytosterols and the fatty acid composition were determined using GC-FID, while tocopherols concentrations were determined using HPLC. The study showed that the quality of seeds used in the production of oil varies. The drying of milk thistle seeds using air cooler than 80 °C caused no statistically significant changes in AV, *p-*AnV, phytosterol levels, tocopherols, or SFA levels. Drying temperatures in the 100–140 °C range caused significant losses of phytosterols and tocopherols and also resulted in changes in fatty acid composition. When seeds were dried at 140 °C, phytosterol levels dropped by 19–23%, tocopherols by 10–23%, MUFA by 30%, and PUFA by 11%.

## Introduction

In some countries of North and Central America, Africa, Australia, and the Middle East, milk thistle is considered a problematic invasive weed (Holm et al. 1997; Montemurro et al. 2007). In contrast, in the countries of Central and Eastern Europe, in Austria, Germany, Egypt, China, and Argentina, milk thistle is cultivated commercially as a medicinal plant. In the Czech Republic in 2010–2013, milk thistle was grown on 4500–5000 ha (Koláčková et al. 2015).

Milk thistle is a herbaceous plant used mainly in the pharmaceutical industry. In recent years, interest in this plant has also increased in the food industry. The strong upward trend in the use of milk thistle is due to the fact that it is a valuable raw material in the production of cooking oil and biofuels, while its by-products, such as oil cake and flours, are used as sources of silymarin and proteins.

Milk thistle was heavily researched in the years 1970–1990; at that time, much effort was put into producing a variety with a high silymarin content. Silymarin flavonolignans have numerous biological activities such as antioxidant, anti-inflammatory and immunomodulatory effects that are connected with its hepatoprotective properties (Polyak et al. 2010). Different milk thistle genotypes have variable levels of silymarin—e.g., the Royston genotype is rich in silymarin (6–10%) (Alemardan et al. 2013).

The milk thistle varieties obtained for this study were not improved in terms of oil or bioactive substance contents, as it is difficult to obtain high-fat seeds in Poland. Polish farmers have focused mainly on the production varieties with high levels of silymarin, which is found in the seed coat.

Thistle seeds are gray-brown, black, shiny, and 5–7 mm long (Andrzejewska et al. 2011). They contain about 25% oil, which consists of 60% linoleic acid and about 20% oleic acid. Proteins make up 25–30%, sterols 0.63%, tocopherols 0.038%, and flavonoids about 2% (Szczucińska et al. 2006).

Phytosterols and tocopherols are important lipid components from a nutritional point of view. The main function of phytosterols is lowering the level of cholesterol in human blood (Moruisi et al. 2006). They also protect against breast, colon and lung cancers (Woyengo et al. 2009; Awad et al. 2007). β-Sitosterol is the most predominant in milk thistle oil, follow campesterol and stigmasterol (Harrabi et al. 2016). Tocopherols (α-, β-, γ- and δ-tocopherols and tocotrienols) have vitamin E activity. α-Tocopherol has become synonymous with vitamin E because it is predominant homologue in human tissues and plays a crucial role in human reproduction, promotes brain health and reverses neurodegeneration, is favorable in lung functions, regulates the expression of immune-related genes in heart, prevents and treats negative cardiovascular diseases outcomes (Ranard and Erdman 2018). α-Tocopherol had the highest amount among the tocopherols detected in milk thistle oil (Fathi-Achachlouei and Azadmard-Damirchi 2009). The content of flavonoids depend on the water availability and environmental conditions during growing of plants (Ghassemi-Golezani et al. 2017).

A serious problem for producers of milk thistle oil is the asynchronous flowering and seed sowing. At the time of harvest, the plants have flower heads at all stages of development, and thus uneven seed maturation (Alemardan et al. 2013). The seed is very important in the production of unrefined cold-pressed oils, as it moisture, taste, smell, and appearance significantly affect the oil parameters. The quality of milk thistle increases with the degree of seed maturation. Immature seeds are smaller, thinner, and relatively lighter-colored than mature seeds. Three different lots seed from different suppliers were dried at 40–140 °C to determine the quality and composition of the milk thistle oils. Drying the seeds was meant to improve their parameters and to allow determination of the effect of the drying temperature on the physical parameters of the oil.

## Materials and methods

### Materials

The experimental material consisted of milk thistle seeds harvested in Poland (PL1 and PL2) and Russia (RS) in 2016. The species of milk thistle was not identified. There was a visible difference in the color of the seeds. Samples RS and PL2 were light colored but black dominated in PL1. RS and PL1 were larger than PL2. All seeds were wetted prior drying by spraying to achieve 14% moisture content, and then stored at 8 °C for 24 h. A moisture content of about 14% is typical for wet summer crops. The control sample was milk thistle seeds, which directly after harvesting were dried up to moisture content of 6%.

### Drying

**Fig. 1:**
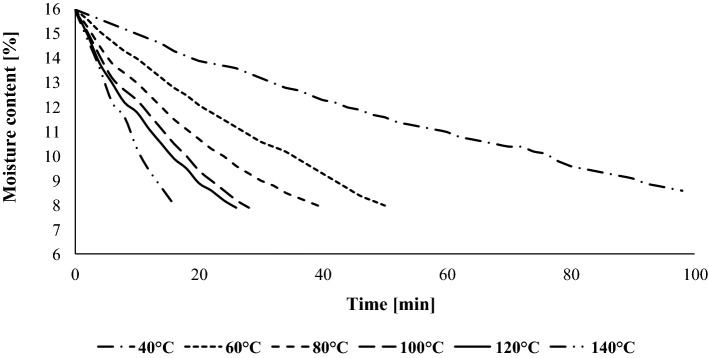
Changes in milk thistle seed moisture during drying at different temperatures

Drying was carried out in a laboratory drier located at the Department of Food Engineering and Industry of the University of Life Sciences in Poznań. The device consisted of two trays composed of drying sieves lined with a thin bed. During the research seeds were automatically weighed and the moisture content was calculated using a computer program based on the material balance. The air was heated to 40 °C, 60 °C, 80 °C, 100 °C, 120 °C and 140 °C. Each seeds variety were dried separately on a thin bed of 0.005 m. Drying was carried out until the seeds reached moisture content of 7%. The tests were performed twice. The seeds were dried for 12 min at 140 °C to 98 min at 40 °C (Fig. 1). The mass of each sample, which were dried was 200 g. Experiment was performed in two replicates.

### Determination of seed moisture content

An electronic moisture analyzer (MA150 Sartorius Mechatronics, Poland) was used to test seed moisture. The reference standard was a 5 g sample dried to a constant mass at 115 °C. The analyzer accuracy was 0.05% wet basis. The AOCS official Method Ba 2a-38 method was used to calibrate the moisture analyzer (AOCS 2007a). All measurements were taken in three repetitions.

### Oil extraction

Oil was extracted from the milk thistle seeds using the procedure described by Folch et al. (1957). Grinding of seeds was carried out in a universal mill (M-20 IKA1-Werke, Germany). The seeds were homogenized 1 min using a chloroform: methanol (2:1 v/v) mixture. Water was used to wash the solvent. To separate the two layers, the mixture was mixed on the vortex for a few seconds and centrifuged at 224 g. A Büchi R 215 rotatory evaporator (Büchi Labortechnik, Flawil, Switzerland) was used to evaporate under vacuum the chloroform layer. Oils were extracted from six autonomous dried samples.

### Acid value (AV)

The official AOCS Cd 3d-63 method was used to determine acid value. The acid value was determined as the amount of KOH (in milligrams) necessary to neutralize the free fatty acids contained in 1 g of oil (AOCS 2007b). Samples from an autonomous samples were analyzed in triplicate.

### Peroxide value (PV)

Peroxide value was determined using the official AOCS method Cd 8b-90. The peroxide value was expressed as milliequivalents peroxide per 1000 g sample. A mixture of acetic acid: chloroform (3:2, v/v) was used to dissolve the oil samples. A saturated KI solution was added to the mixture. The mixture was shaken for 1 min and deionized water was added. The obtained sample was titrated using 0.002 M Na_2_S_2_O_3_ (AOCS 2011a).

### *p*-Anisidine value (*p*-AnV)

The value of *p*-anisidine was determined using the official AOCS method Cd 18-90. Isooctane was used to dilute the sample. The absorbance of the solution at 350 nm was then measured using a UV-1800 spectrophotometer (Shimadzu, Kyoto, Japan) (AOCS 2011b).

### Fatty acid composition

Fatty acid methyl esters (FAME) were generated using AOCS official Method Ce 1k-07. Separation of the diluted FAME was performed in the HP 5890 II series (Hewlett Packard, Palo Alto, CA, USA) equipped with a flame ionization detector and Innowax capillary column (30 m × 0, 20 mm × 0.20 mm). The carrier gas used was hydrogen at a flow rate of 1.5 mL/min. The initial temperature of the column was 60 °C and was increased by 12 °C/min to 200 °C, then the temperature was maintained for 15 min. The injector and detector temperature was 240 °C. Fatty acids were identified by comparing their retention times with exterior those of standards. Standards fatty acids were purchased from Sigma-Aldrich (USA). The results were obtained after integration and calculation using Chem. Station (Agilent Technologies) as weight percentages (AOCS 2007c).

### Tocopherol contents

Tocopherols were separated and quantified by HPLC according to AOCS method Ce 8-89 (AOCS 1997a). Saponification of seeds samples (2 g) was performed using 60% KOH (2 mL), ethanol (20 mL) and pyrogallol (0.5 g) at the boiling point of ethanol (78 °C) for 30 min. Unsaponifiable substances were extracted with 50 mL of *n*-hexane/ethyl acetate (90:10 v/v). High-performance liquid chromatography (Waters 600 Milford, MA, USA) was used in 100 mL unsaponifiable organic layer. A LiChrosorb Si60 column (250 mm × 4.6 mm × 5 μm) and the LiChrospher Si60 preliminary column were used for the test. A mobile phase was a mixture of *n*-hexane and 1,4-dioxane (97: 3 v/v) with a flow rate of 1.5 mL/min. The excitation of the fluorometric detector (Waters 474 Milford, MA, USA) was 290 nm and the emission 330 nm (Gawrysiak-Witulska et al. 2011). Identification of all tocopherols (α-T, β-T, γ-T, δ-T) consisted in a comparison of retention times. Concentrations were calculated by comparing the obtained surfaces with surfaces from authentic tocopherol standards (Merck, Darmstadt, Germany).

### Sterol contents

Sterol contents were determined using the official AOCS method 6-91 (AOCS 1997b) with the modification of Ciftci et al. (2011). Extracted oils (0.05 g) were saponified with 1 M KOH in methanol. The unsaponifiables were extracted with a mixture of hexane and methyl-tert-butyl ether (1:1, v/v). Solvent was evaporated and dry phase was silylated using Sylon BTZ (Supelco, Bellefonte, PA, USA). The sterol derivatives were separated on an HP 6890 gas chromatograph equipped with a DB-35MS capillary column (25 m × 0.20 mm × 0.33 µm, J&W Scientific, Folsom, CA, USA). Samples were injected in splitless mode. Column temperature was programmed: 100 °C for 5 min, followed by the increase rate: 25 °C/min to 250 °C. The temperature of 250 °C was maintained for 1 min and then the increase rate was programmed: 3 °C/min to 290 °C and held for 20 min. Injector and detector temperature was set at 300 °C. A carrier gas was hydrogen at a flow rate of 1.5 mL/min. Internal standard was 5α-cholestan used to quantify sterol. Phytosterols were identified using Agilent Technologies 7890A GC coupled to the V75 MSD triple axis 5975C detector after separation on a DB-5 capillary column (30 m × 0.2 mm × 0.32 μm, J & W Scientific, Folsom, CA, USA) and calculated by comparison of the received data with the standard. Mass spectra were recorded using electron ionization at 70 eV and scanning mass in the range of 100–650 Da. The carrier gas was helium with a flow rate of 0.6 mL/min. The temperature of the ion source was maintained at 200 °C and the injector at 300 °C. Mass spectra were identified on the basis of the NIST Mass Spectra Library and laboratory spectrum library of sterols.

### Statistical analysis

Each result shown in the tables and figures presents the mean of three replicate analyses for each sample ± the standard deviations. Results are presented using Statistica 10.0 (StatSoft, Tulsa, OK, USA). The value of p < 0.05 means significant differences between the mean values obtained as a result of the analysis of variance. The differences between mean values were determined by analysis of variance (ANOVA), and the post-hoc analysis was performed using Tukey’s test.

## Results and discussion

**Fig. 2:**
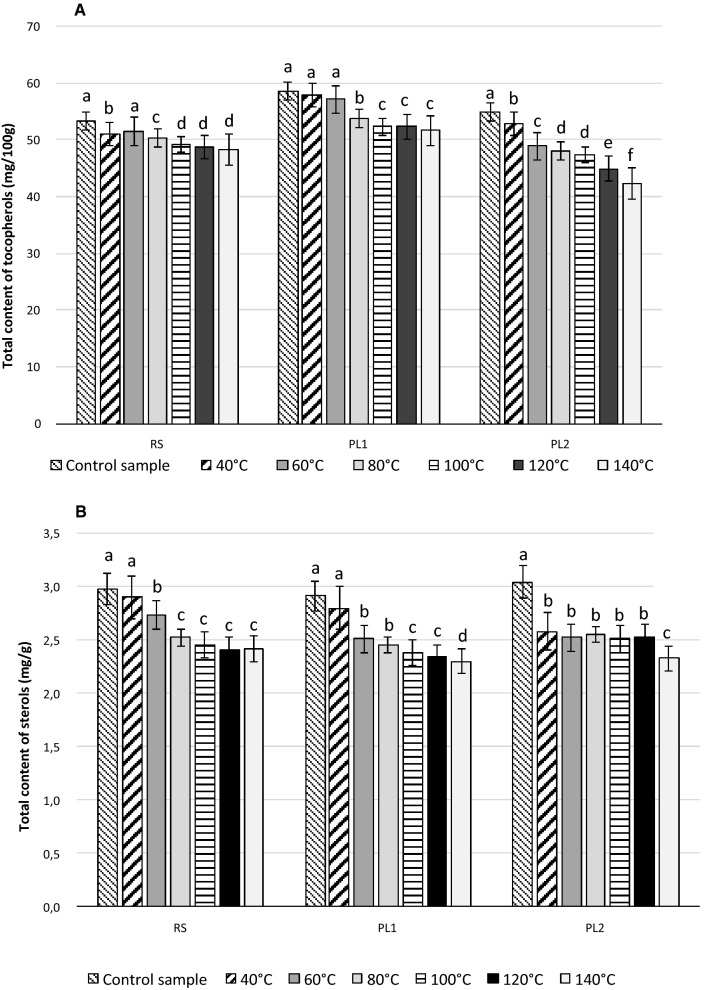
The changes of tocopherol (**a**) and sterol (**b**) contents in oils extracted from fresh and dried milk thistle seeds

**Fig. 3:**
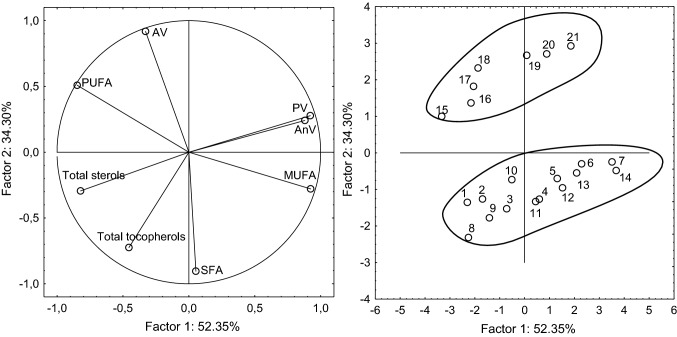
Principal component analysis (PCA) of the loadings plot and the score plot of data from total tocopherols, total sterols, saturated fatty acids (SFA), monounsaturated fatty acids (MUFA), polyunsaturated fatty acids (PUFA), acid value (AV), peroxide value (PV), anisidine value (*p*-AnV) in oils extracted from milk thistle seeds RS (1–7), PL1 (8–14) and PL2 (15–21)

The PV, AV, *p*-AnV, fatty acid composition, and phytosterol and tocopherol contents were determined for the oils extracted from dried and undried milk thistle seeds. The results are shown in Tables 1, 2, 3 and Figs. 1, 2, 3.

**Table 1:** Acid value, peroxide value, *p*-anisidine value and fatty acid composition of lipids extracted from milk thistle seeds dried at different temperatures

Samples of seeds	Acid value (mg KOH/g)	Peroxide value (meq O_2_/kg)	*p*-Anisidine value	C16:0 (%)	C18:0 (%)	C18:1 (%)	C18:2 (%)	C18:3 (%)	C20:0 (%)	C20:1 (%)	Others (%)
RS											
Control sample	0.95 ± 0.00^a^	3.48 ± 0.05^ g^	0.01 ± 0.00^e^	7.3 ± 0.4^b^	0.3 ± 0.1^a^	21.2 ± 0.8^c^	63.4 ± 1.1^a^	0.5 ± 0.0^a^	4.1 ± 0.2^a^	3.0 ± 0.2^a^	0.3 ± 0.1^b^
40 °C	0.92 ± 0.00^a^	7.91 ± 0.04^f^	0.01 ± 0.00^e^	7.5 ± 0.4^b^	0.3 ± 0.0^a^	21.8 ± 0.7^c^	63.1 ± 1.3^a^	0.4 ± 0.0^a^	4.1 ± 0.1^a^	2.2 ± 0.2^b^	0.6 ± 0.2^a^
60 °C	0.94 ± 0.00^a^	8.30 ± 0.03^e^	0.20 ± 0.01^d^	7.8 ± 0.3^a^	0.3 ± 0.1^a^	22.8 ± 0.8^c^	62.4 ± 1.1^a^	0.4 ± 0.0^a^	4.1 ± 0.2^a^	2.2 ± 0.1^b^	0.1 ± 0.0^c^
80 °C	0.93 ± 0.00^a^	9.52 ± 0.08^d^	0.50 ± 0.03^c^	7.7 ± 0.3^a^	0.3 ± 0.1^a^	24.0 ± 0.6^b^	61.3 ± 1.0^a^	0.4 ± 0.1^a^	4.0 ± 0.3^a^	2.1 ± 0.1^b^	0.3 ± 0.0^b^
100 °C	0.92 ± 0.00^a^	12.53 ± 0.10^c^	0.60 ± 0.10^c^	7.8 ± 0.4^a^	0.3 ± 0.1^a^	24.8 ± 0.8^a^	60.9 ± 0.9^b^	0.3 ± 0.1^a^	3.5 ± 0.3^b^	1.9 ± 0.2^b^	0.5 ± 0.1^a^
120 °C	0.94 ± 0.00^a^	15.89 ± 0.11^b^	1.28 ± 0.12^b^	7.6 ± 0.2^a^	0.3 ± 0.0^a^	25.2 ± 0.9^a^	60.7 ± 0.8^b^	0.3 ± 0.0^a^	3.5 ± 0.2^b^	1.9 ± 0.2^b^	0.4 ± 0.1^a^
140 °C	0.94 ± 0.00^a^	20.73 ± 0.13^a^	1.79 ± 0.15^a^	7.7 ± 0.3^a^	0.3 ± 0.1^a^	26.3 ± 0.6^a^	60.0 ± 0.8^b^	0.1 ± 0.0^b^	3.4 ± 0.2^b^	1.9 ± 0.1^b^	0.4 ± 0.1^a^
PL1											
Control sample	1.68 ± 0.00^a^	2.00 ± 0.01^ g^	0.30 ± 0.04^d^	8.8 ± 0.3^a^	0.2 ± 0.0^a^	21.6 ± 0.6 ^d^	61.6 ± 1.1^a^	1.3 ± 0.2^a^	3.1 ± 0.1^a^	2.4 ± 0.3^a^	1.1 ± 0.1^b^
40 °C	1.68 ± 0.00^a^	7.99 ± 0.04^f^	0.59 ± 0.08^c^	8.7 ± 0.4^a^	0.2 ± 0.1^a^	21.5 ± 0.5^d^	61.4 ± 1.2^a^	1.2 ± 0.2^a^	3.0 ± 0.3^a^	2.4 ± 0.2^a^	1.8 ± 0.4^a^
60 °C	1.68 ± 0.00^a^	8.32 ± 0.02^e^	0.59 ± 0.06^c^	8.3 ± 0.3^b^	0.2 ± 0.0^a^	22.9 ± 0.6^c^	61.8 ± 0.9^a^	1.2 ± 0.1^a^	2.8 ± 0.3^a^	2.3 ± 0.2^a^	0.7 ± 0.3^c^
80 °C	1.68 ± 0.00^a^	10.70 ± 0.06^d^	0.69 ± 0.10^c^	9.0 ± 0.4^a^	0.1 ± 0.0^a^	23.8 ± 0.7^c^	61.0 ± 0.9^a^	1.2 ± 0.2^a^	2.9 ± 0.2^a^	1.9 ± 0.2^b^	0.2 ± 0.1^d^
100 °C	1.68 ± 0.00^a^	11.16 ± 0.07^c^	1.29 ± 0.11^b^	9.0 ± 0.4^a^	0.1 ± 0.0^a^	24.8 ± 0.7^b^	59.7 ± 0.9^b^	1.1 ± 0.1^a^	2.5 ± 0.2^b^	1.5 ± 0.3^b^	1.2 ± 0.3^b^
120 °C	1.68 ± 0.00^a^	15.59 ± 0.10^b^	1.48 ± 0.11^b^	8.8 ± 0.3^a^	0.1 ± 0.0^a^	25.3 ± 0.6^b^	59.6 ± 0.5^b^	0.9 ± 0.2^b^	2.4 ± 0.1^b^	1.6 ± 0.2^b^	1.3 ± 0.2^b^
140 °C	1.68 ± 0.00^a^	23.44 ± 0.14^a^	1.61 ± 0.08^a^	8.9 ± 0.3^a^	0.1 ± 0.0^a^	26.7 ± 0.6^a^	58.9 ± 0.6^b^	0.9 ± 0.1^b^	2.4 ± 0.1^b^	1.6 ± 0.2^b^	0.5 ± 0.1^c^
PL2											
Control sample	8.95 ± 0.01^a^	3.24 ± 0.02^e^	0.01 ± 0.00^c^	7.1 ± 0.3^b^	0.1 ± 0.0^b^	21.7 ± 0.5^c^	64.1 ± 1.1^a^	0.7 ± 0.1^a^	3.5 ± 0.2^a^	2.0 ± 0.1^a^	0.9 ± 0.2
40 °C	8.98 ± 0.00^a^	6.34 ± 0.04^d^	0.01 ± 0.00^c^	7.4 ± 0.4^b^	0.1 ± 0.0^b^	21.7 ± 0.5^c^	64.1 ± 1.1^a^	0.6 ± 0.1^a^	3.5 ± 0.3^a^	1.9 ± 0.2^a^	0.9 ± 0.2^b^
60 °C	8.96 ± 0.01^a^	6.39 ± 0.05^d^	0.01 ± 0.00^c^	7.5 ± 0.4^b^	0.1 ± 0.0^b^	21.4 ± 0.6^c^	64.1 ± 0.8^a^	0.6 ± 0.0^a^	3.5 ± 0.3^a^	1.5 ± 0.3^b^	1.4 ± 0.3^a^
80 °C	8.97 ± 0.01^a^	6.44 ± 0.05^d^	0.01 ± 0.00^c^	7.6 ± 0.3^b^	0.1 ± 0.0^b^	22.5 ± 0.7^b^	63.9 ± 0.9^a^	0.6 ± 0.1^a^	2.8 ± 0.3^b^	1.0 ± 0.2^c^	1.4 ± 0.4^a^
100 °C	8.97 ± 0.01^a^	15.06 ± 0.11^c^	1.22 ± 0.10^b^	7.8 ± 0.3^a^	0.2 ± 0.0^a^	23.3 ± 0.8^a^	63.1 ± 0.5^b^	0.6 ± 0.1^a^	2.6 ± 0.2^b^	1.0 ± 0.1^c^	1.5 ± 0.3^a^
120 °C	8.97 ± 0.01^a^	17.10 ± 0.10^b^	1.78 ± 0.11^a^	7.9 ± 0.2^a^	0.3 ± 0.1^a^	23.9 ± 0.7^a^	63.2 ± 0.9^b^	0.5 ± 0.1^b^	2.7 ± 0.2^b^	0.8 ± 0.2^c^	0.7 ± 0.3^b^
140 °C	8.97 ± 0.01^a^	19.24 ± 0.12^a^	1.87 ± 0.11^a^	7.9 ± 0.3^a^	0.5 ± 0.1^a^	24.4 ± 0.7^a^	63.1 ± 0.9^b^	0.5 ± 0.0^b^	2.7 ± 0.2^b^	0.8 ± 0.2^c^	0.1 ± 0.1^c^

Data represents means ± SDs (standard deviation) (n = 3). Values with different superscript letters within each column for individual samples are significantly different at p < 0.05

**Table 2:** Tocopherol contents of oils extracted from fresh and dried milk thistle seeds (mg/100 g of oil)

Samples of seeds	α-Tocopherol	β-Tocopherol	γ-Tocopherol	δ-Tocopherol
RS				
Control sample	46.68 ± 0.24^a^	2.53 ± 0.11^a^	3.76 ± 0.12^a^	0.37 ± 0.01^a^
40 °C	45.51 ± 0.06^b^	2.49 ± 0.03^a^	3.60 ± 0.08^b^	0.35 ± 0.01^a^
60 °C	45.21 ± 0.06^ab^	2.47 ± 0.04^a^	3.52 ± 0.09^b^	0.35 ± 0.01^a^
80 °C	44.32 ± 0.05^c^	2.46 ± 0.04^a^	3.42 ± 0.02^b^	0.35 ± 0.01^a^
100 °C	42.96 ± 0.08^d^	2.44 ± 0.01^a^	3.43 ± 0.01^b^	0.34 ± 0.01^b^
120 °C	42.80 ± 0.06^d^	2.33 ± 0.03^b^	3.25 ± 0.05^c^	0.34 ± 0.01^b^
140 °C	42.78 ± 0.03^d^	2.04 ± 0.06^c^	3.05 ± 0.03^d^	0.33 ± 0.00^b^
PL1				
Control sample	50.39 ± 0.35^a^	3.69 ± 0.01^a^	3.99 ± 0.01^a^	0.50 ± 0.03^a^
40 °C	49.84 ± 0.01^b^	3.64 ± 0.02^a^	3.98 ± 0.01^a^	0.47 ± 0.01^a^
60 °C	49.69 ± 0.16^b^	3.36 ± 0.05^b^	3.66 ± 0.01^b^	0.46 ± 0.01^a^
80 °C	46.47 ± 0.16^c^	3.23 ± 0.04^c^	3.62 ± 0.05^b^	0.44 ± 0.00^b^
100 °C	45.22 ± 0.06^d^	3.17 ± 0.04^c^	3.50 ± 0.07^c^	0.43 ± 0.01^b^
120 °C	45.14 ± 0.04^d^	3.19 ± 0.01^c^	3.53 ± 0.02^c^	0.43 ± 0.01^b^
140 °C	44.77 ± 0.17^c^	3.17 ± 0.04^c^	3.26 ± 0.07^d^	0.40 ± 0.01^c^
PL2				
Control sample	47.78 ± 0.14^a^	2.03 ± 0.04^a^	4.74 ± 0.04^a^	0.32 ± 0.00^a^
40 °C	46.18 ± 0.10^b^	2.03 ± 0.02^a^	4.42 ± 0.04^b^	0.31 ± 0.01^a^
60 °C	42.50 ± 0.01^c^	1.92 ± 0.08^ab^	4.17 ± 0.07^c^	0.31 ± 0.01^a^
80 °C	41.96 ± 0.10^d^	1.84 ± 0.01^b^	3.97 ± 0.02^d^	0.29 ± 0.01^a^
100 °C	41.34 ± 0.13^de^	1.82 ± 0.01^b^	3.91 ± 0.02^d^	0.28 ± 0.02^ab^
120 °C	39.29 ± 0.06^e^	1.80 ± 0.02^b^	3.54 ± 0.12^e^	0.25 ± 0.01^b^
140 °C	37.20 ± 0.08^f^	1.59 ± 0.09^c^	3.20 ± 0.13^f^	0.24 ± 0.01^b^

Values are means ± standard deviation (n = 3). Different superscript letters in columns for each sample indicate statistically significant differences at the level p = 0.05

**Table 3:** Sterol contents of oil extracted from milk thistle seeds before and after drying at different temperatures (mg/g of oil)

Samples of seeds	Phytosterols (mg/g)							
	Campesterol	Δ5-Stigmasterol	β-Sitosterol	Δ5-Avenasterol	Δ7-Stigmasterol	Δ7-Avenasterol	Cholesterol	Others
RS								
Control sample	0.22 ± 0.01^a^	0.27 ± 0.02^a^	1.38 ± 0.06^a^	0.05 ± 0.01^a^	0.76 ± 0.05^a^	0.12 ± 0.01^a^	0.12 ± 0.01^a^	0.06 ± 0.01^a^
40 °C	0.19 ± 0.02^b^	0.25 ± 0.03^a^	1.36 ± 0.06^a^	0.06 ± 0.01^a^	0.78 ± 0.02^a^	0.12 ± 0.01^a^	0.10 ± 0.01^b^	0.04 ± 0.00^b^
60 °C	0.19 ± 0.01^b^	0.24 ± 0.02^a^	1.24 ± 0.05^a^	0.06 ± 0.01^a^	0.75 ± 0.04^a^	0.12 ± 0.01^a^	0.09 ± 0.01^b^	0.04 ± 0.00^b^
80 °C	0.17 ± 0.01^c^	0.22 ± 0.02^b^	1.16 ± 0.06^b^	0.06 ± 0.01^a^	0.68 ± 0.02^b^	0.11 ± 0.01^a^	0.09 ± 0.01^b^	0.04 ± 0.01^b^
100 °C	0.16 ± 0.01^c^	0.23 ± 0.02^b^	1.10 ± 0.05^b^	0.05 ± 0.00^a^	0.66 ± 0.01^b^	0.13 ± 0.01^a^	0.09 ± 0.01^b^	0.03 ± 0.00^c^
120 °C	0.16 ± 0.02^c^	0.21 ± 0.03^b^	1.09 ± 0.06^b^	0.05 ± 0.00^a^	0.65 ± 0.03^b^	0.12 ± 0.01^a^	0.09 ± 0.00^b^	0.03 ± 0.00^c^
140 °C	0.17 ± 0.02^c^	0.21 ± 0.02^b^	1.09 ± 0.05^b^	0.05 ± 0.01^a^	0.65 ± 0.01^b^	0.13 ± 0.01^a^	0.09 ± 0.01^b^	0.03 ± 0.00^c^
PL1								
Control sample	0.19 ± 0.01^a^	0.25 ± 0.02^a^	1.21 ± 0.04^a^	0.08 ± 0.02^a^	0.60 ± 0.04^a^	0.15 ± 0.02^a^	0.37 ± 0.02^a^	0.06 ± 0.01^a^
40 °C	0.17 ± 0.01^b^	0.25 ± 0.01^a^	1.13 ± 0.04^b^	0.07 ± 0.01^a^	0.60 ± 0.02^a^	0.15 ± 0.02^a^	0.38 ± 0.02^a^	0.05 ± 0.01^a^
60 °C	0.15 ± 0.01^c^	0.22 ± 0.01^b^	0.97 ± 0.06^c^	0.07 ± 0.01^a^	0.55 ± 0.02^b^	0.14 ± 0.01^a^	0.36 ± 0.01^a^	0.05 ± 0.00^a^
80 °C	0.15 ± 0.01^c^	0.21 ± 0.01^b^	0.96 ± 0.03^c^	0.06 ± 0.00^a^	0.54 ± 0.02^b^	0.13 ± 0.01^b^	0.36 ± 0.01^a^	0.04 ± 0.00^b^
100 °C	0.13 ± 0.01^d^	0.20 ± 0.02^b^	0.95 ± 0.04^c^	0.06 ± 0.01^a^	0.52 ± 0.01^c^	0.13 ± 0.01^b^	0.35 ± 0.01^b^	0.04 ± 0.00^b^
120 °C	0.14 ± 0.01^d^	0.19 ± 0.01^c^	0.93 ± 0.03^c^	0.05 ± 0.00^b^	0.52 ± 0.01^c^	0.13 ± 0.02^b^	0.34 ± 0.01^b^	0.04 ± 0.00^b^
140 °C	0.13 ± 0.01^d^	0.19 ± 0.01^c^	0.94 ± 0.01^c^	0.05 ± 0.01^b^	0.50 ± 0.01^c^	0.12 ± 0.01^b^	0.33 ± 0.01^c^	0.03 ± 0.00^c^
PL2								
Control sample	0.27 ± 0.01^a^	0.35 ± 0.02^a^	1.39 ± 0.06^a^	0.08 ± 0.01^a^	0.60 ± 0.03^a^	0.16 ± 0.01^a^	0.15 ± 0.00^a^	0.04 ± 0.01^a^
40 °C	0.22 ± 0.01^b^	0.30 ± 0.02^b^	1.14 ± 0.04^b^	0.07 ± 0.01^a^	0.52 ± 0.02^b^	0.15 ± 0.01^a^	0.14 ± 0.01^a^	0.04 ± 0.00^a^
60 °C	0.22 ± 0.01^b^	0.29 ± 0.01^b^	1.14 ± 0.04^b^	0.07 ± 0.01^a^	0.50 ± 0.02^b^	0.14 ± 0.01^b^	0.13 ± 0.01^b^	0.03 ± 0.00^b^
80 °C	0.21 ± 0.01^b^	0.30 ± 0.01^b^	1.14 ± 0.03^b^	0.07 ± 0.02^a^	0.51 ± 0.02^b^	0.14 ± 0.01^b^	0.13 ± 0.00^b^	0.05 ± 0.00^a^
100 °C	0.22 ± 0.01^b^	0.29 ± 0.01^b^	1.12 ± 0.03^b^	0.07 ± 0.01^a^	0.51 ± 0.02^b^	0.14 ± 0.01^b^	0.13 ± 0.00^b^	0.03 ± 0.00^b^
120 °C	0.22 ± 0.01^b^	0.29 ± 0.01^b^	1.11 ± 0.03^b^	0.07 ± 0.01^a^	0.52 ± 0.02	0.14 ± 0.01^b^	0.13 ± 0.01^b^	0.04 ± 0.00^b^
140 °C	0.20 ± 0.01^c^	0.25 ± 0.01^c^	1.10 ± 0.03^b^	0.05 ± 0.00^b^	0.48 ± 0.01^c^	0.12 ± 0.01^c^	0.10 ± 0.01^c^	0.03 ± 0.01^b^

Values are means ± standard deviations (n = 3). Different superscript letters in columns for each sample indicate statistically significant differences at the level p = 0.05

### Oils quality

The levels of AV in the oils extracted from the undried milk thistle seeds were 0.95 mg KOH/g oil for RS, 1.68 for PL1, and 8.95 for PL2 seeds (Table 1). The high AV of the oil from the PL2 seeds disqualified it as a food product, on the basis of the Codex Alimentarius (2005) requirements for cold-pressed oils (AV < 4). The AV of cold-pressed milk thistle seed oils originating from Tunisia has been shown to range from 5.48 to 8.34 mg KOH/g (Meddeb et al. 2017). The high AV is associated with the free fatty acids formed during enzymatic hydrolysis in the seeds, which they can cause the oxidation of oils during production and storage.

Drying the seeds did not cause statistically significant changes in the acid value (Table 1). In another study, no changes in AV were observed when yellow-seeded *Brassica napus* seeds were dried at 40 °C and 60 °C, but after drying at 80–120 °C the AV increased from 1.33 mg KOH/g to 1.68 mg KOH/g (Gawrysiak-Witulska et al. 2015). Malekzadeh et al. (2011) demonstrated that drought stress affected the AV of milk thistle seed oil, with oil from nonirrigated plants having lower AVs.

The peroxide values (PV) of oils extracted from fresh seeds were low: 2.0 meq O_2_/kg for PL1, 3.24 meq O_2_/kg for PL2, and 3.48 meq O_2_/kg for RS (Table 1). These results are in accordance with those reported by Meddeb et al. (2017). An increase in drying temperature caused an increase in peroxide value. For cold-pressed oils, the Codex Alimentarius (2005) specifies a maximum peroxide value of 15 meq O_2_/kg. All oils extracted from the dried seeds at 120 °C and 140 °C showed PV > 15 meq O_2_/kg.

The secondary oxidation products were measured by *p*-AnV and the results showed that the levels of these compounds in the oils from undried seeds were very low, ranging from 0.01 (RS and PL2) to 0.3 (PL1) (Table 1). There are no standardized maximum *p-*AnV limits for good raw edible vegetable oil. The *p*-AnV increased with drying temperature. The highest increases were found after drying at 100 °C and 140 °C (1.6–1.9). All samples had *p*-AnV below 2. Casal et al. (2010) found *p*-AnV = 6 in extra virgin olive oil, 3 and 4 in virgin olive oils, and 6 in refined olive oil.

### Fatty acids composition

Table 1 shows the average fatty acid compositions of the samples. Air-drying of the seeds at temperatures below 80 °C caused no statistically significant changes in percentage composition of PUFA. When the drying temperature increased to 100–140 °C, the share of PUFA in total fatty acids decreased. Linoleic acid was the most abundant of the polyunsaturated fatty acids in all the oils, and varied from 63% (control sample) to 60% (dried at 140 °C) in the RS oil, from 62% (control sample) to 59% (dried at 140 °C) in the PL1 oil, and from 64% (control sample) to 63% (dried at 100 °C) in the PL2 oil. Oleic acid was the most common monounsaturated fatty acid in all the oils, ranging from 21% (control sample) to 26% (dried at 140 °C) in the RS oil, from 22% (control sample) to 27% (dried at 140 °C) in the PL1 oil, and from 22% (control sample) to 24% (dried at 120 °C) in the PL2 oil. Palmitic acid was the most abundant of the saturated fatty acids, and varied between 7 and 9%, while arachidic acid ranged from 2 to 4%, depending on the sample and the drying temperature (Table 1). The most visible changes in fatty acid composition were seen in oils extracted from seeds PL1 and PL2 dried at 100–140 °C range. The fatty acid composition of oils obtained from citrus seeds dried at 60–80 °C was not significant different from the control samples, but a large increase in palmitic acid and decrease in oleic acid percentage was seen (Al Juhaimi et al. 2018). In the study of Gawrysiak-Witulska et al. (2007, 2015), insignificant changes in the fatty acid composition of oils extracted from yellow-seed and black-seeded *Brassica napus* were seen. Krasucki et al. (2002) detected significant changes in the fatty acid composition during the drying of rapeseeds at 80–180 °C.

### Tocopherols

**Fig. 4:**
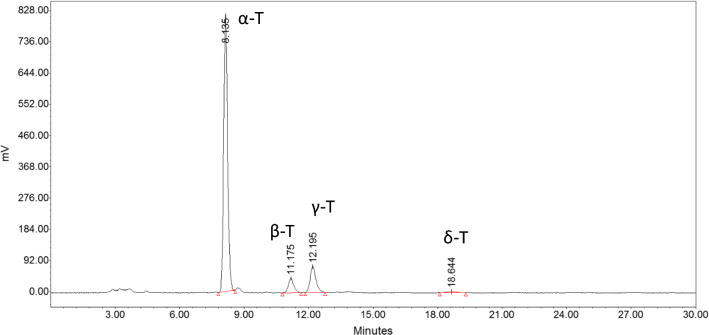
HPLC chromatogram of tocopherols in the tested seeds of milk thistle

Four homologues: α-, β-, γ- and δ-tocopherols were identified in the milk thistle seed oils (Fig. 4). The levels of individual tocopherols are shown in Table 2 and their total levels are given in Fig. 2a. Milk thistle seeds have a similar tocopherol content as rapeseeds. The total tocopherols in the control samples constituted 53.34–58.57 mg/100 g of the oil. The dominant tocopherol in rapeseed is the γ-T homologue (Gawrysiak-Witulska et al. 2015), in milk thistle seeds the dominant tocopherol was α-T (46.68–50.39 mg/100 g), which made up 86% of the total tocopherol content. The β-T, γ-T, and δ-T contents were 2.03–3.69 mg/ 100 g (6%), 3.76–4.42 mg/ 100 g (7%), and 0.32–0.50 mg/ 100 g (1%), respectively. Similar results for the individual tocopherols were shown by Fathi-Achachlouei and Azadmard-Damirchi (2009), where α-T constituted 86–87% of the total tocopherols. In our study, the total tocopherol content decreased during drying of the milk thistle seeds (Table 2, Fig. 2a). In oil extracted from the RS, PL1, and PL2 seeds dried at 40 °C, the total tocopherol losses were respectively 3%, 1%, and 4%; at 60 °C, they decreased respectively by 3%, 2%, and 11%; at 80 °C, by 5%, 8% and 12%; at 100 °C, by 8%, 11% and 14%; at 120 °C, by 9%, 11% and 18%; and at 140 °C, by 10%, 12% and 23%. The tocopherol losses in two samples were similar, but in the third oil, they were twice as great. It may be the result of the seed quality, and associated with the smaller losses of α-T. The decrease in the content of this homologue in the RS and PL1 oils ranged from 3% (at 40 °C) to 8% (at 140 °C), and from 1% (at 40 °C) to 11% (at 140 °C), respectively. For the PL2 oil, the losses of α-T were higher, and ranged from 3% (at 40 °C) to 22% (at 140 °C).

During drying at 40 °C and 60 °C, losses of β-, γ- and δ-T homologues did not exceed 7%, with the highest losses being found in the PL2 oil. Increasing the air temperature to 80 °C led to losses of these compounds of 3–9% (for RS), 9–12% (for PL1), and 9–16% (for PL2). During drying at 120 °C, the losses were 8–11% of β-T, 12–25% of γ-T, and 8–22% of δ-T. Drying at 140 °C led to losses of β-T tocopherol homologues of 19% for RS, 14% for PL1, and 22% for PL2. The losses of γ-T were similar for RS and PL1, and amounted to 18%, while in the PL2 sample it reached as high as 32%. Analysis of this index showed that the losses of tocopherols during drying in the RS and PL1 oils were similar, while losses were greater in the PL2 oil. Generally, drying the seeds at different temperatures led to decreases in both the total amount and the individual homologues of tocopherols. When citrus seeds were dried at 80 °C, the content of tocopherols in the extracted oils decreased by about 50% (Al Juhaimi et al. 2018).

### Phytosterols

The changes in the total phytosterol contents in the oils extracted from milk thistle seeds dried at different temperatures are presented on Fig. 2b, while those of the individual sterols are given in Table 3. In the control samples, the total sterol contents ranged from 2.91 to 3.04 mg/g of oil. This was half the size of the value given by Ismaili et al. (2016), who found 6.27 mg/g of sterols in oil extracted from seeds of the Moroccan milk thistle. Studies by Fathi-Achachlouei and Azadmard-Damirchi (2009) have shown that the level of phytosterols in different varieties of milk thistle from Iran ranged from 1.80 to 2.20 mg/g. The composition of the sterol fraction in the tested seeds was typical for milk thistle oil. Six major plant phytosterols: campesterol, stigmasterol, β-sitosterol, avenasterol, Δ7-stigmasterol and Δ7-avenasterol, along with cholesterol, were found in the investigated oils.

The dominant sterols were β-sitosterol (1.21–1.39 mg/g), followed by Δ7-stigmasterol (0.60–0.76 mg/g), with both accounting for 42–46% and 20–26% of the total sterol content, respectively. The other sterols were found in much lower amounts: Δ5-stigmasterol was at 0.25–0.35 mg/g, campesterol at 0.19–0.27 mg/g, and cholesterol at 0.12–0.37 mg/g; they constituted respectively 9–12%, 7–9%, and 4–13% of the total sterols. The individual sterol contents and their percentage compositions were in agreement with the results of studies of Harrabi et al. (2016) and Fathi-Achachlouei and Azadmard-Damirchi (2009). In all dried samples, a decrease was observed in the total sterol contents (Fig. 2B). In the RS and PL1 seeds dried at 40 °C, the losses of total sterols were 3 and 4%, respectively; however, these differences were not statistically significant. Much greater losses (15%) were recorded during drying of the PL2 sample at the same temperature. During drying at 80 °C, the total sterol losses amounted to 15–16% in all oils; they were 17–18% at 100 °C, but increased to 19–23% at 140 °C.

Individual sterols were also degraded by the drying conditions (Table 3). When drying was performed at 40 °C, 60 °C, and 80 °C the degradation of campesterol was more rapid than of other sterols, amounting to 11–19%, 14–21% and 21–23%, respectively. When drying at 100 °C, this sterol degraded 19–32%, and at this temperature the most rapid degradation was observed for all individual sterols. The highest decrease (of 38%) was seen for avenasterol in oils extracted from the PL1 and PL2 seeds dried at 140 °C. Avenasterol has one double bond on the ring part of its molecule and an ethylidene group in the side chain, which can affect its rate of degradation. Gawrysiak-Witulska et al. (2015) showed that avenasterol degraded more rapidly during drying of yellow-seeded rapeseed.

### Statistical analysis

Principal component analysis (PCA) was used to observe clusters in the milk thistle seeds. The first two main factors comprised 86.65% (PF1 = 52.35% and PF2 = 34.30%) of total variation. The PCA results showed noticeable differences between the milk thistle samples (Fig. 3). Factor 1 was mainly correlated with MUFA content (r = 0.924), PV (r = 0.921), and *p*-AnV (0.880). It was also negatively correlated with PUFA (r = − 0.848), and total sterol content (r = − 0.822). Factor 2 was mainly correlated with acid value (r = 0.918) and negatively correlated with SFA content (r = − 0.905) and total tocopherols (r = − 0.724). The data shown in the score plot divided the samples into two groups.

All oil samples extracted from the RS (samples 1–7) and PL1 seeds (samples 8–14) are located below the *x*-axis. Above the *x*-axis are the oil samples from the PL2 seeds (samples 8–14). Oils extracted from control and dried RS and PL1 seeds were characterized by lower AV than those from PL2 seeds. Additionally, during drying, significant decreases in PUFA and increases in MUFA occurred. All samples of oils from the control seeds and from those seeds dried at lower temperatures (40 °C, 60 °C), as well as PL2 oils from seeds dried at 80 °C, are located on the left of the plot. These samples were characterized by higher PUFA levels, total sterols, and tocopherol levels. The oils extracted from the RS and PL1 seeds dried at 80 °C, 100 °C, 120 °C and 140 °C, and from the PL2 seeds dried at 100 °C, 120 °C, and 140 °C were located on the right side of plot. These sample showed higher levels of MUFA, PV, and *p*-AnV.

## Conclusion

Milk thistle oil is produced by small manufacturers using cold pressing, and is used as an unconventional oil rich in bioactive compounds. Our study has shown that the quality of seeds used for production of oil can vary. Of the three analyzed samples, one (PL2) had very high AV which in practice disqualified it as a raw material for oil production, though the composition was otherwise unproblematic.

Air-drying of the seeds at temperatures below 80 °C caused no statistically significant changes in AV, *p*-AnV or phytosterol, tocopherol, and SFA contents. Increasing the drying temperature to 100–140 °C led to significant losses of phytosterol and tocopherol contents and changes in fatty acid composition. When seeds were dried at 140 °C, 19–23% of phytosterols, 10–23% of tocopherols, 30% of MUFA, and 11% of PUFA were lost. Generally, high-quality seeds are needed if high-quality oils are to be produced. The exposure to heat during the drying process decreased the amount of bioactive compounds and accelerated oxidation.

## Abbreviations

AV: Acid value
T: Tocopherol
α-T: Alpha-tocopherol
β-T: Beta-tocopherol
γ-T: Gamma-tocopherol
δ-T: Delta-tocopherol
MUFA: Monounsaturated fatty acids
PUFA: Polyunsaturated fatty acids
SFA: Saturated fatty acids
